# Ultrasound treatment enhances tomato drought resistance from germination to seedling stage

**DOI:** 10.3389/fpls.2026.1738812

**Published:** 2026-02-10

**Authors:** António Nogueira, António Teixeira, Joana Martins, Hernâni Gerós, Hélder Puga

**Affiliations:** 1 CMEMS–UMinho - Centre for Microelectromechanical Systems, University of Minho, Guimarães, Portugal; 2 CBMA–UMinho - Centre of Molecular and Environmental Biology, Department of Biology, University of Minho, Braga, Portugal; 3 CEB–UMinho - Centre of Biological Engineering, Department of Engineering, University of Minho, Braga, Portugal

**Keywords:** antioxidant capacity, drought stress, seed germination, seedling vigor, ultrasound treatment

## Abstract

Drought stress poses a major threat to tomato seed germination and early seedling growth. These critical stages for the establishment of a successful crop are compromised by drought, which disrupts water uptake, impairs metabolic functions, and induces oxidative damage. As a cornerstone of global agriculture, tomato (*Solanum lycopersicum*) is increasingly vulnerable to these environmental pressures during its early development stages. With climate change amplifying the frequency and intensity of droughts, these challenges are growing, thereby threatening agricultural productivity worldwide. This study investigates the efficacy of ultrasound-assisted seed treatment as a novel strategy to mitigate drought effects, using osmotic stress induced by D-mannitol (ranging from 0 to –0.75 MPa). Tomato seeds were treated with multifrequency and multimode ultrasound technology (19.8 kHz, 200 W, 7.5 min) and evaluated for germination, seedling vigor, and biochemical responses under controlled conditions. Results demonstrate that ultrasound significantly enhances the germination percentage (up to 34% at –0.75 MPa) and seedling emergence (up to 36% at –0.50 MPa), while reducing germination time by up to 17% under high osmotic stress (–0.50 and –0.75 MPa). Moreover, ultrasound significantly increased the seedling vigor index I and chlorophyll content (up to 50% at −0.50 MPa). Superoxide dismutase and catalase activity in seeds increased by up to 45% and 77%, respectively, with similar values observed in seedlings. Total antioxidant activity (DPPH, ABTS, FRAP) increased in seeds and seedlings by up to 70% (under all conditions). Ultrasound-treated seeds exhibited elevated malondialdehyde (MDA) levels (up to 31% at –0.75 MPa), indicating an initial mechanical stress, while the resulting seedlings showed a reduction of MDA in all conditions (up to 48%), suggesting enhanced membrane stability over time. Principal component analysis and correlation analysis revealed distinct physiological and biochemical trait variations, with ultrasound effects more pronounced in seedlings. These findings highlight ultrasound’s potential to activate adaptive mechanisms, counteracting drought-induced oxidative damage and improving early plant performance. This scalable, eco-friendly technique offers a promising alternative to conventional priming methods.

## 1 Introduction

Tomato (*Solanum lycopersicum*) is a widely consumed fruit (commonly treated as a vegetable in culinary contexts) that plays a crucial role in human nutrition. It is the most widely grown vegetable in the global production system, with an annual production of approximately 186 million tons reported in 2022 (Tiwari et al., 2022). Tomatoes are rich in nutrients and bioactive compounds, including proteins, amino acids, fatty acids, vitamin C, potassium, folic acid, carotenoids, and dietary fiber (Kumar et al., 2021; Collins et al., 2022).

Drought, water availability, temperature, salinity, and environmental contaminants are key factors influencing crop performance, significantly affecting plant growth, metabolism, and overall yield. Drought is the most devastating abiotic stress, significantly hindering global agricultural productivity (Kopecká et al., 2023). Its prevalence is exacerbated by global climate change, leading to excessive evapotranspiration, unpredictable precipitation patterns, and prolonged periods of water scarcity, thereby threatening sustainable crop yield and quality worldwide (Li et al., 2009; Lobell et al., 2011). For example, drought-induced economic losses in the EU and UK have been estimated at approximately €9 billion, with up to 60% of these losses attributed to the agricultural sector in certain regions (Cammalleri et al., 2020). This impact is expected to escalate in the coming decades due to the intensifying effects of climate change and forecasts indicating that by 2050, nearly half of the world’s arable land will face drought stress (Kaya et al., 2006; Marthandan et al., 2020).The germination phase and early seedling growth are particularly vulnerable under drought conditions, disrupting key physiological and metabolic processes (Saha et al., 2022). Crops such as tomato (Ishtiaq et al., 2023), rice (Ali et al., 2004) and wheat (Duan et al., 2025) have shown significant declines in germination rates and seedling vigor when exposed to water-deficient environments. Prolonged drought stress disrupts critical physiological processes, including photosynthesis, nutrient uptake and translocation (e.g., nitrogen, phosphorus, potassium), ultimately reducing plant height, leaf area, and root proliferation. Furthermore, drought-induced oxidative stress generates excessive reactive oxygen species (ROS), such as hydroxyl radicals and hydrogen peroxide, causing lipid peroxidation, membrane damage, DNA and protein damage, and loss of cellular function. These cascading effects culminate in reduced crop yields and compromised quality, posing severe challenges for global food security, particularly in rainfed agricultural regions (Sharma et al., 2012; Tabassum et al., 2018; Saha et al., 2022).

Priming, an alternative to conventional breeding, includes techniques such as hydropriming, osmopriming, and chemical or hormonal priming, which can improve seed stress tolerance and enhance germination by reducing hydration time (Marthandan et al., 2020). Studies have shown that priming activates protective mechanisms against oxidative stress, increases enzyme activity, boosts metabolite production, and repairs DNA damage (Hussain et al., 2016; Zheng et al., 2016; Marthandan et al., 2020; Aswathi et al., 2022). For instance, a study on drought-stressed tomato seeds demonstrated significant improvement in plant growth, chlorophyll content, and enzymatic activity of superoxide dismutase (SOD) and catalase (CAT), while reducing ROS and lipid peroxidation (Ishtiaq et al., 2023). Despite their potential, these techniques often require significant time and labor, and show variable effectiveness across different priming methods, crops and drought conditions (Ashraf and Foolad, 2005; Srivastava et al., 2021; Tu et al., 2022). This scenario has motivated interest in innovative methods such as ultrasound-assisted priming, which offers a more efficient and reliable alternative for improving seed quality and potentially boosting stress tolerance (Nogueira et al., 2023).

Ultrasound technology presents a scalable, cost-effective, and eco-friendly solution for improving seed performance and promoting sustainable agricultural practices. By generating high-intensity acoustic waves, ultrasound induces mechanical and cavitation effects that improve seed coat permeability and activate metabolic and biochemical processes that stimulate germination and seedling vigor (Nogueira et al., 2023, 2025). Research has demonstrated ultrasound’s ability to improve seed hydration and upregulate genes involved in water transport (aquaporins), as well as in hormone regulation (gibberellin and salicylic acid). Additionally, ultrasound has been shown to stimulate key enzymatic activities, including those of antioxidant enzymes (SOD and CAT) and starch hydrolysis (via *α*-amylase and *β*-amylase), as well as nutrient mobilization, while minimizing the production of ROS and reducing malondialdehyde (MDA) levels (Yaldagard et al., 2008; Silva et al., 2020; Alfalahi et al., 2022; Gong et al., 2024; Nogueira et al., 2025). These metabolic and biochemical alterations have been shown to improve germination rates as well as seedling growth and vigor across various crops (Nogueira et al., 2023). For example, a study on tomato reported a 43% increase in germination percentage, a 19% faster germination rate, enhanced seedling vigor, and reduced seedling emergence time, highlighting ultrasound as a promising alternative seed priming strategy (Nogueira et al., 2024).

Beyond tomato, increasing evidence indicates that ultrasound treatment can mitigate the adverse effects of drought or osmotic stress in other crop species. Ultrasound has been shown to improve the germination of castor bean seeds exposed to varying concentrations of polyethylene glycol 8000 (PEG) (Babaei et al., 2023) and to reduce oxidative damage in pepper seedlings by decreasing hydrogen peroxide and malondialdehyde accumulation while enhancing antioxidant enzyme activity under drought stress (Yakupoğlu, 2023). However, to the best of our knowledge, the application of ultrasound seed treatment to enhance drought tolerance in tomato has not yet been investigated. Moreover, the temporal dynamics of oxidative stress responses induced by ultrasound from seeds to developing seedlings remain poorly understood.

The present study aims to evaluate the potential of ultrasound treatment to mitigate drought stress effects on tomato seed germination and early seedling performance. Additionally, the study investigates the underlying physiological and biochemical mechanisms, particularly oxidative defense responses, and their persistence into seedling development. To address these objectives, tomato seeds were subjected to simulated drought conditions using varying osmotic potentials of D-mannitol under *in vitro* conditions.

## 2 Materials and methods

### 2.1 Seed materials

Commercially available seeds of tomato (*Solanum lycopersicum* var. cerasiforme) were purchased from a local market. The seeds were stored in a dry place at 20°C until use.

### 2.2 Ultrasound processing

#### 2.2.1 Ultrasound instrumentation

The seed processing experiments were conducted on a laboratory scale using multifrequency, multimode, and modulated (MMM) technology (Nogueira et al., 2024). This system included a piezoelectric transducer, a booster, an acoustic waveguide, and a probe specifically designed and optimized for the system’s functionality, featuring oscillation capabilities that allow various vibration modes and harmonics of ultrasound frequencies to be exhibited. MMM technology activates multiple vibratory modes within the acoustic load, promoting a uniform distribution of high-intensity acoustic energy. This design overcomes traditional efficiency limitations by preventing the formation of stationary standing wave patterns.

#### 2.2.2 Ultrasound seed treatment

For the ultrasound treatment, continuous pulse trains were applied using the entire ultrasound unit with the probe immersed 2 cm into the medium (a water-seed mixture). The treatment was carried out at a frequency of 19.8 kHz with a ±100 Hz sweep and a power output of 200 W, parameters optimized based on previous work (Nogueira et al., 2024). This operating window falls within the low-frequency ultrasound range, in which acoustic cavitation is the principal mechanism underlying biological effects, providing a controlled and biologically safe treatment optimized for reproducibility and mechanistic analysis. The tests were conducted in a glass flask with specific dimensions (0.2 cm wall thickness, 17.0 cm height, 6.0 cm inner diameter). Seed samples were suspended in 500 mL of sterilized distilled water, and the solution was sonicated for 7.5 min. To ensure a uniform dispersion of ultrasound waves across all seeds and minimize overexposure, the seeds and liquid were continually stirred using a magnetic stirrer set at 250 rpm during the treatment. The experiments were conducted in a temperature-controlled room set at 16°C. Temperature consistency between the treatment and control groups was ensured by regular monitoring. To minimize overheating, cooled water was occasionally refilled in both groups. The control seeds were soaked in the same volume of water for the same amount of time as the treated seeds but were not sonicated.

### 2.3 Drought stress

Following ultrasound treatment, drought stress was induced during germination using D-mannitol at varying concentrations (100, 200, and 300 mM) to create three levels of osmotic stress (Ψ_w_ = –0.25, –0.50, and –0.75 MPa, respectively) (Píriz-Pezzutto et al., 2025). A treatment condition with no added D-mannitol in the agar acted as the 0 MPa control. Germination was carried out by sowing the seeds in transparent resealable plastic boxes (17 × 13.5 × 9.5 cm) filled with 1% (w/v) agar, half-strength Murashige and Skoog basal medium and the various osmotic conditions.

### 2.4 Germination experiments

The experiments were conducted in triplicate, with each replicate consisting of 50 tomato seeds. Seeds were incubated at 24°C under an 8-hour dark/16-hour light photoperiod (200 *μ*mol photons m^−2^ s^−1^). Germination was monitored daily for 14 days with seeds considered germinated once the radicles were visibly protruding and reaching a length of 2 mm. Additionally, some tomato seeds were germinated for only two days before being stored at -80°C for further experiments whereas other tomato seeds were stored ungerminated (T_0_). Upon completion of the seedling growth period, the root and shoot lengths, and the fresh and dry weight were measured (average weight of 20 seedlings). Moreover, various germination and seedling parameters were determined. The final germination percentage (GP) and seedling emergence (SE) indicate the total number of seeds that have successfully germinated and the total number of seedlings that have emerged from the germinated seeds by the end of the test, respectively. GP and SE were calculated as follows, Equations 1, 2:

(1) $$GP\;\left(\%\right)=\;\frac{Germinated\;seeds}{Total\;number\;of\;seeds}\;\times 100$$

(2) $$SE\;\left(\%\right)=\;\frac{Seedlings\;emerged}{Total\;number\;of\;germinated\;seeds}\;\times 100$$

The germination value (GV) was calculated as a composite index that takes into account both the speed and the total germination percentage of the seeds (Czabator, 1962), with higher values indicating more vigorous seed performance. The GV was calculated as follows, Equation 3:

(3) $$GV=\;\frac{\sum DGS}{N}\;\times GP\;\times 10$$

where, DGS represents the number of newly germinated seeds per day and *N* represents the total number of seeds.

The time to 50% germination (T_50_) and the time to 25% germination (T_25_, included in –0.75 MPa conditions, as germination did not reach 50% values) were determined as indicators of germination speed and calculated using Equation 4 (Coolbear et al., 1984):

(4) $$T_{50}\;\left(days\right)=\;ti+\;\frac{\left[\left(\frac{N}{2}\right)-ni\right]\;\times \left(tj-ti\right)}{\left(nj-ni\right)}$$

where, *N* represents the total number of seeds, *ti* is the time when the cumulative germination reached less than *N/2*; *tj* is the time in which the cumulative germination exceeded *N/2*; *ni* represents the cumulative number of seeds germinated at time *ti* and *nj* is the cumulative number of seeds germinated at time *tj.*

The seedling vigor index I (SVI I) provides an overall measure of seedling size and robustness, taking into account both the germination capacity and the growth of the seedlings, while the seedling vigor index II (SVI II) focuses on the accumulation of dry matter in the seedlings, which is an indicator of the seedlings’ ability to convert stored reserves into biomass during early growth. The indices were calculated using the following Equations 5, 6:

(5) $$\text{SVI I }=\frac{\left(\text{Root length}+\text{Shoot length}\right)\times \text{GP}}{100}$$

(6) $$\text{SVI II}=\frac{\text{Seedling dry weight}\times \text{GP}}{100}$$

### 2.5 Crude extraction

After the germination period, seeds and seedlings were immediately frozen in liquid nitrogen and ground to a fine powder using a pre-chilled mortar and pestle. The frozen samples were then lyophilized for 3 days (Christ Alpha 2–4 LD Plus lyophilizer). Following the freeze-drying process, the completely dehydrated samples were stored in a desiccator for subsequent analyses.

#### 2.5.1 Sample extractions for enzymatic assays

For the antioxidant enzymes, each group of seeds (100 mg) and seedlings (5 mg) was homogenized in 5 and 2.5 mL of 0.1 M sodium phosphate buffer (pH 7.5) containing 1% (w/v) polyvinylpyrrolidone and 0.5 mM ethylenediaminetetraacetic acid (EDTA), respectively. The homogenate was then centrifuged at 5000 g for 30 min at 4°C. The resulting supernatant was collected for the enzymatic assays of CAT and SOD activities.

#### 2.5.2 Sample extractions for antioxidant assays

Each group of seeds (50 mg) and seedlings (5 mg) was homogenized in 1 mL of 80% methanol. The homogenate was then centrifuged at 5000 g for 30 min at 4°C. The resulting supernatant was collected for the antioxidant activity assays (DPPH, ABTS and FRAP assays).

### 2.6 Chlorophyll and carotenoids content

Chlorophyll content was measured following the method of Curtis and Shetty (1996). In brief, 2 mg of dry seedling samples were homogenized in 1 mL of methanol (90%). After filtration, the solutions were incubated in the dark to prevent photo-oxidation. Chlorophyll *a* (Chl *_a_*) and chlorophyll *b* (Chl *_b_*) contents were quantified by measuring the absorbance at 665.2 nm (A_665.2_) and 652.4 nm (A_652.4_), respectively. Carotenoid content was determined at 470 nm (A_470_). Calibration was performed using 90% methanol as a blank. The Chl *_a_*, Chl *_b_*, total chlorophyll (Chl *_a_*_+_*_b_*) and total carotenoids concentration (Car *_c_*) (µg/mg dry matter) were calculated using the following equations Equations 7–10 (Lichtenthaler, 1987).

(7) $$Chl_{a}=16.82A_{665.2}-9.28A_{652.4}$$

(8) $$Chl_{b}=36.92A_{652.4}-16.54A_{665.2}$$

(9) $$Chl_{\;a+b}=0.28A_{665.2}-27.64A_{652.4}$$

(10) $$Car_{\;c}=\;\;\frac{1000A_{470}-1.91Chl_{a}-95.15Chl_{b}}{225}$$

### 2.7 Lipid peroxidation – MDA content

Lipid peroxidation was assessed by measuring the MDA content following the method described by Cakmak and Horst (1991). Lyophilized seed and seedling samples were homogenized in 1.2 mL of 0.1% (w/v) trichloroacetic acid, followed by centrifugation at 12,000 *g* for 10 min. A 300 *µ*L aliquot of the supernatant was then mixed with an equal volume of 0.5% (w/v) thiobarbituric acid. The mixture was heated at 95°C for 30 min, and the reaction was stopped by immediately placing the tubes on ice. After cooling for 5 min, the tubes were centrifuged again at 12,000 *g* for 10 min. The absorbance of the supernatant was measured at 532, 600, and 450 nm to determine the MDA content. Interference from soluble sugars at 532 nm and 450 nm was corrected by subtraction. The MDA content (µmol/g DW) was calculated using the Equation 11:

(11) $$\left[MDA\right]\;=\;6.45\;\times \;\left(A_{532}\;-\;A_{600}\right)\;-\;0.56\;\times \;A_{450}$$

where, A532, A600 and A450 represent the absorbance of the mixture at 532, 600, and 450 nm, respectively.

### 2.8 Antioxidant enzyme activity

#### 2.8.1 SOD activity

The SOD activity was determined following the method described by Vuleta et al. (2016), which is based on the enzyme’s ability to inhibit the photoreduction of nitro blue tetrazolium (NBT). The reaction mixture contained 50 mM sodium phosphate buffer (pH 7.4), 50 *µ*M NBT, 0.1 mM EDTA, 50 mM sodium carbonate, 12 mM L-methionine, 10 *µ*M riboflavin, and 20 µL of crude extract, with a final volume of 300 *µ*L. A reaction mixture without the crude extract was used as the blank control. The reaction was initiated by exposing the mixture to white light for 30 min at room temperature, after which the absorbance was measured at 560 nm. One unit (U) of SOD activity was defined as the amount of enzyme causing 50% inhibition of the photochemical reduction of NBT. The SOD activity was expressed as U/mg of dry weight.

#### 2.8.2 CAT activity

CAT activity was measured following the method described by Aebi (1984). Briefly, the reaction mixture contained 100 mM sodium phosphate buffer (pH 7.0), 30 mM H_2_O_2_, and 40 *µ*L of crude extract, with a total volume of 200 *µ*L per well. The activity was monitored spectrophotometrically at 30°C by tracking the absorbance decrease at 240 nm, corresponding to the decomposition of H__2__O__2__. The CAT activity was expressed as *μ*mol of H_2_O_2_ oxidized per min per gram of dry weight.

### 2.9 Total antioxidant activity

#### 2.9.1 DPPH radical scavenging assay

Antioxidant activity was assessed using the DPPH (1,1-diphenyl-2-picrylhydrazyl) radical scavenging assay, following the method proposed by Baliyan et al. (2022). Dry seed and seedling samples were mixed with 0.4 mM DPPH in 80% methanol and thoroughly shaken. The mixture was incubated in the dark at room temperature for 30 min. After incubation, absorbance was measured at 517 nm using methanol as the blank. The DPPH radical scavenging activity of the extracts was calculated using the following Equation 12:

(12) $$DPPH\;scavenging\;activity\;\left(\%\right)=\frac{\left(Abs_{control}\;-\;Abs_{sample}\right)}{Abs_{control}}\;\times 100$$

where, Abs_control_ indicates the absorbance value of the reaction mixture without sample extracts and Abs_sample_ represents the absorbance of the reaction mixture with sample extracts.

#### 2.9.2 ABTS radical scavenging assay

The 2,2′-azino-bis(3-ethylbenzothiazoline-6-sulfonic acid) (ABTS) radical cation decolorization assay was carried out following the method of Youn et al. (2019) with minor modifications. The ABTS+ solution was prepared by mixing 2.45 mM potassium persulfate with 7 mM ABTS+ in distilled water at a 1:1 ratio. The mixture was stored at room temperature in the dark for 16 h to allow the formation of the ABTS radical cation. Before use, the solution was diluted with 90% methanol to achieve an absorbance of 0.700 at 734 nm. The sample methanolic extracts were then mixed with the diluted ABTS+ solution and incubated in the dark for 5 min before measuring the absorbance at 734 nm. The ABTS radical scavenging activity was calculated using Equation 13:

(13) $$ABTS\;radical\;scavenging\;activity\;\left(\%\right)=\frac{\left(Abs_{C}\;-\;Abs_{S}\right)}{Abs_{C}}\;\times 100$$

where, Abs_c_ refers to the absorbance at 734 nm of the blank and Abs_s_ refers to the absorbance at 734 nm of the different samples analyzed.

#### 2.9.3 Ferric reducing antioxidant power assay

The antioxidant capacity, based on the reduction of the Fe^3+^ -2,4,6-tri(2-pyridyl)-s-triazine (TPTZ) complex (colorless) to Fe^2+^ tripyridyltriazine (blue), was measured (Ferreira-Santos et al., 2022). Briefly, the FRAP reagent was prepared by combining 300 mM sodium acetate buffer (pH 3.6), 10 mM TPTZ in 40 mM hydrochloric acid, and 20 mM FeCl_3_ in a 10:1:1 ratio at 37°C. Sample methanolic extracts were mixed with the FRAP reagent and incubated for 30 min. The absorbance was measured at 593 nm using a blank (with no added extract) as the reference. A calibration curve was plotted using a stock solution of Trolox (2 mg/mL in 80% methanol) and diluted to concentrations ranging from 0.1 to 0.5 mM. Results were expressed as mM of Trolox equivalents per g of dry sample.

### 2.10 Statistical analysis

Data reported in the figures were analyzed using a two-tailed Student’s t-test for single comparisons using Prism 10 (GraphPad Software, Inc., La Jolla, CA, USA). Differences between the control group and the ultrasound treatment were assessed using a paired t-test at *p* < 0.05. Results are expressed as means ± standard error (n = 3). Plots were marked with asterisks to denote the significance level as compared to those of the control: *p < 0.05; **p < 0.01; ***p < 0.001. Principal Component Analysis was performed using the FactoMineR package for R.

## 3 Results

### 3.1 Contribution of ultrasound treatment to the physiological and biochemical variability

**Figure 1:**
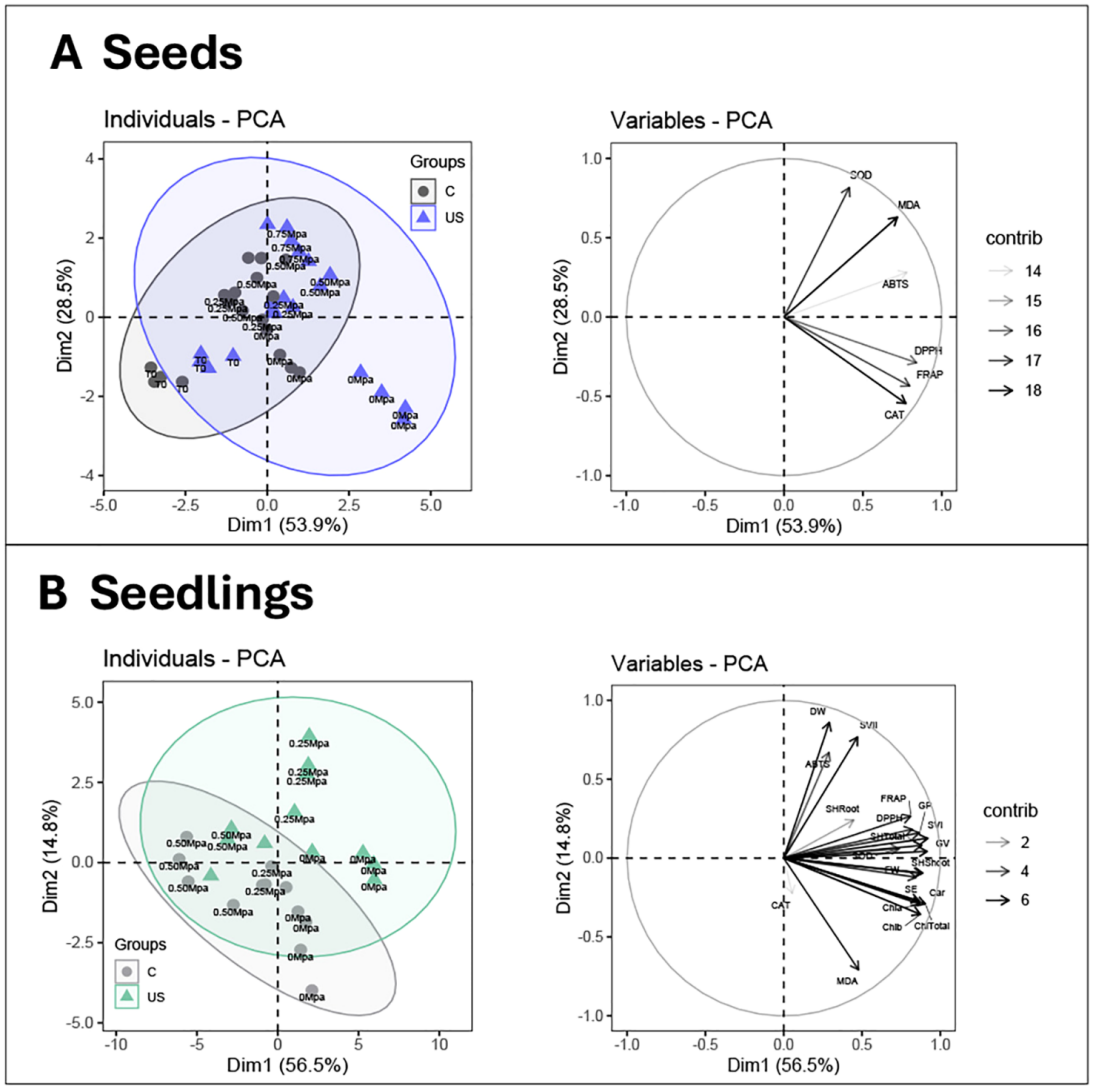
Principal component analysis of physiological and biochemical parameters in tomato seeds **(A)** and seedlings **(B)** treated with ultrasound (US) and control (C) under different osmotic potentials (0; –0.25; –0.50; –0.75 MPa). Left: Individual factor map showing the distribution of each treatment at different osmotic potential. Ellipses represent 95% confidence intervals. Right: Variable factor map displaying the correlation between measured parameters and their contribution to principal components. Arrow length indicates the contribution strength of each variable to the principal components. Variables measured include: antioxidant enzymes (SOD, superoxide dismutase; CAT, catalase), oxidative stress marker (MDA, malondialdehyde), antioxidant capacity (DPPH, ABTS, FRAP), seedling growth parameters (GP, germination percentage; GV, germination value; SE, seedling emergence; SVI-SVII, seedling vigor indices I and II; seedling heights (SHRoot:root; SHShoot: shoot; SHTotal:total (root + shoot)); FW: fresh weight; DW: dry weight), and photosynthetic pigments (Chl _a_, chlorophyll a; Chl _b_, chlorophyll b; Chl _a + b_, total chlorophyll; Car, carotenoids).

PCA of tomato seeds subjected to osmotic stress and treated with ultrasound showed a generally low impact on the physiological and biochemical traits compared to the control group (**Figure 1A**). This effect was more pronounced in the tomato seedlings under the same conditions (**Figure 1B**). The first two principal components accounted for a cumulative variance of 82%, and while both control and ultrasound treatments were distributed along the first dimension (Dim 1; 53%), osmotic pressure effects were explained by Dim 2, which accounted for 29% of the variability (**Figure 1A**, left). The variable factor map of seeds revealed a strong positive correlation of traits with Dim 1, particularly MDA and CAT, indicating their central role in seed responses under osmotic stress (**Figure 1A**, right).

The PCA of tomato seedlings explained a cumulative variance of 71.3%, indicating that the ultrasound treatment was primarily accounted for by Dim 1 whereas the control seedlings were predominantly influenced by Dim 2, accounting for 15% (**Figure 1B**, left). Ultrasound-treated samples clustered separately from controls which indicates an overall enhancement of growth and antioxidant-related traits. The variable factor map for seedlings further clarified the significant contribution of the physiological and biochemical traits along the positive axis of Dim 1, except for root (SHRoot) and CAT (**Figure 1B**, right).

### 3.2 Effects of ultrasound treatment on germination and seedling performance

**Figure 2:**
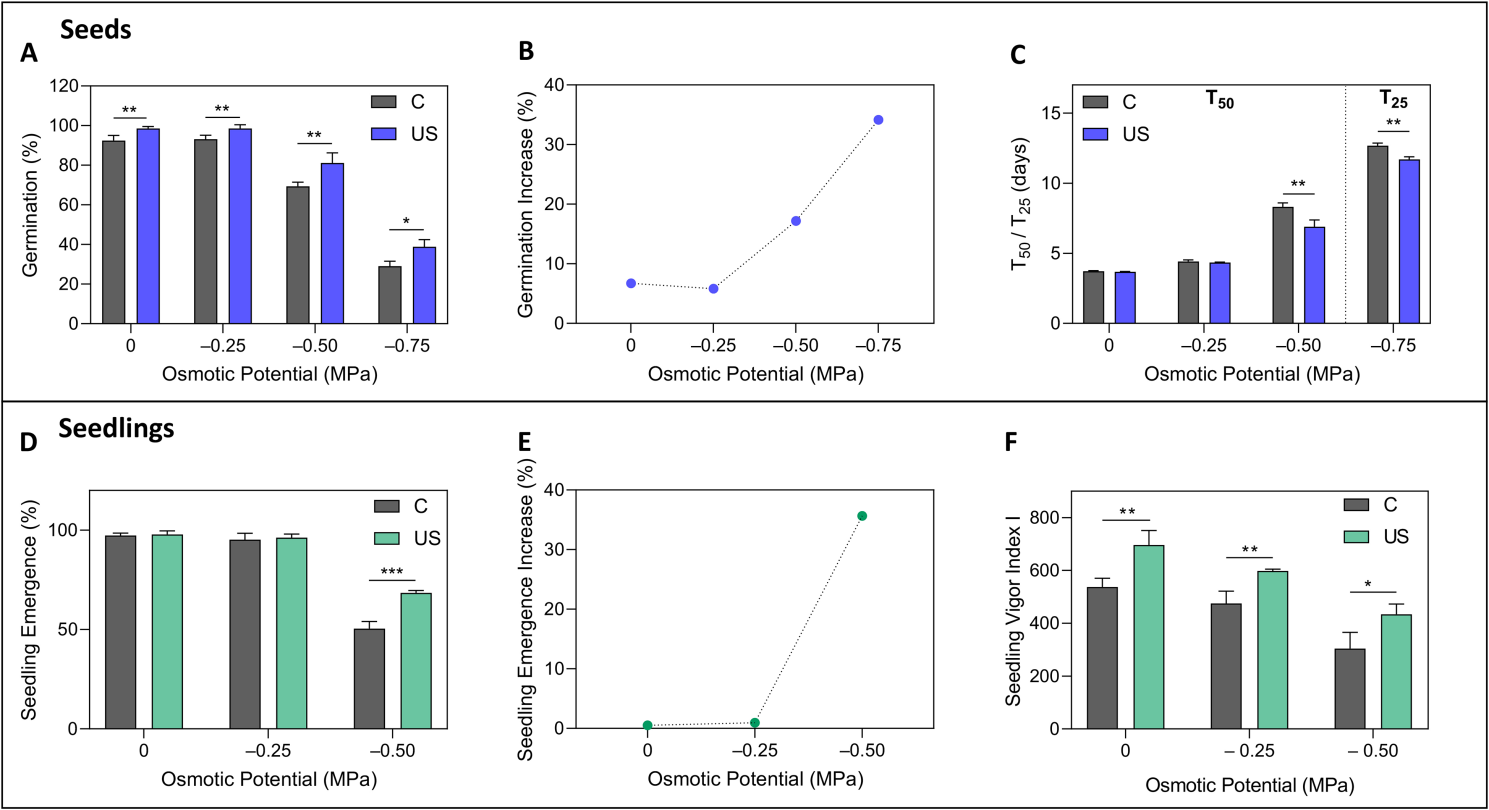
Effects of ultrasound treatment on seed germination and seedling performance under osmotic stress. Germination parameters include final germination percentage **(A)**, germination increase following ultrasound treatment **(B)**, and time to 50% germination (T_50_; C) in control **(C)** and ultrasound-treated (US) seeds across osmotic potentials (0 to –0.75 MPa). Seedling performance parameters include emergence percentage **(D)**, emergence increase with ultrasound treatment **(E)**, and seedling vigor index I [SVI I; **(F)**] under osmotic stress from 0 to –0.50 MPa. Data are presented as means ± SE (n=3). Asterisks indicate significant differences between treatments for each osmotic potential: *p< 0.05; **p< 0.01; ***p< 0.001. Statistical significance was assessed using independent Student’s t-tests.

Ultrasound-treated seeds under osmotic stress exhibited a significantly higher germination percentage than control seeds, achieving an increase of 34% at –0.75 MPa (**Figure 2A**). The beneficial effect of ultrasound was further highlighted when the increase in germination was represented as a function of osmotic pressure (**Figure 2B**). A notable acceleration in germination was evident under high osmotic stress conditions (–0.50 and –0.75 MPa). This enhancement led to a reduction in germination time (i.e., T_50_ and T_25_) of up to 17%, representing 1.2 fewer days compared to the control (**Figure 2C**). The germination value additionally reinforced the positive impact of ultrasound, with treated seeds displaying consistently higher values, except at –0.50 MPa (**Supplementary Figure S1**).

Tomato seedlings from ultrasound-treated seeds and the control group exhibited emergence values close to 100% at lower stress levels (0 and –0.25 MPa). However, an increase of 36% in emergence was noted at –0.50 MPa (**Figure 2D**). This improvement was more apparent when the rise in emergence (%) was depicted as a function of osmotic pressure (**Figure 2E**). Moreover, ultrasound treatment significantly increased seedling vigor index I by up to 43% across all osmotic potentials (**Figure 2F**). Likewise, seedling vigor index II more than doubled at –0.25 MPa following ultrasound treatment (**Supplementary Figure S2**).

### 3.3 Effects of ultrasound treatment on seedling development, biomass accumulation and photosynthetic pigments

Ultrasound treatment mitigated the negative effects of osmotic stress on seedling traits. Specifically, root length increased significantly under non-stressed conditions (0 MPa), while shoot length showed significant improvements by up to 22% at both 0 and –0.50 MPa. These enhancements contributed to a significant increase in total seedling length of up to 24% at 0 and –0.25 MPa. Biomass accumulation was also affected by the osmotic stress gradient, however, ultrasound-treated seedlings exhibited greater stress tolerance and maintained higher fresh and dry weights, with an increase of up to 111%, in the latter compared with the control at –0.25 MPa (**Table 1**).

**Table 1:** Effects of ultrasound treatment on seedling growth parameters under osmotic stress.

Treatments		Seedling height (cm)			Fresh weight (mg)	Dry weight (mg)
		Root	Shoot	Total		
C	[0 MPa]	3.1 ± 0.3	2.7 ± 0.2	5.8 ± 0.3	288.6 ± 15.0	13.0 ± 4.1
US	[0 MPa]	3.8 ± 0.5 *	3.3 ± 0.2 **	7.2 ± 0.6 *	358.5 ± 45.0 *	21.0 ± 0.8 *
C	[0.25 MPa]	3.2 ± 0.2	2.3 ± 0.3	5.3 ± 0.2	241.2 ± 36.1	13.7 ± 0.6
US	[0.25 MPa]	3.5 ± 0.3	2.5 ± 0.2	6.0 ± 0.1 *	258.9 ± 12.6	28.9 ± 3.9 **
C	[0.50 MPa]	2.9 ± 0.6	1.6 ± 0.1	4.4 ± 0.8	144.1 ± 24.6	15.4 ± 4.6
US	[0.50 MPa]	3.6 ± 0.5	1.8 ± 0.1 *	5.4 ± 0.6	176.4 ± 7.5 *	18.4 ± 2.4

Seedling height measurements showing root, shoot, and total seedling length (root + shoot) of control (C) and ultrasound-treated (US) seedlings under different osmotic potentials (0-0.50 MPa). Biomass accumulation expressed as fresh and dry weight (mg). Data are presented as means ± SE (n=3). Asterisks indicate significant differences between treatments for each osmotic potential: *p ≤ 0.05; **p ≤ 0.01. Statistical significance was assessed using independent Student’s t-tests.

The quantification of photosynthetic pigments revealed distinct responses to ultrasound treatment under varying osmotic stress conditions. For instance, ultrasound treatment significantly increased the concentrations of all chlorophylls by up to 50% in seedlings subjected to the highest osmotic stress (–0.50 MPa). Although carotenoid content exhibited a trend towards higher levels in ultrasound-treated seedlings, particularly at 0 MPa, no statistical differences were found (**Table 2**).

**Table 2:** Biochemical responses of seedlings to ultrasound treatment under osmotic stress conditions.

Treatments		Chlorophyll content (μg/mg)			Carotenoids car _c_ (μg/mg)
		Chl _a_	Chl _b_	Total chl _(a+b)_	
C	[0 MPa]	5.6 ± 0.4	1.9 ± 0.2	7.6 ± 0.6	1.4 ± 0.1
US	[0 MPa]	7.4 ± 1.5	2.2 ± 0.5	10.4 ± 0.7 **	1.9 ± 0.4
C	[0.25 MPa]	4.2 ± 0.5	1.5 ± 0.2	5.7 ± 0.3	1.0 ± 0.1
US	[0.25 MPa]	4.0 ± 0.3	1.3 ± 0.1	5.3 ± 0.3	1.0 ± 0.1
C	[0.50 MPa]	2.1 ± 0.2	0.6 ± 0.1	2.7 ± 0.3	0.6 ± 0.1
US	[0.50 MPa]	2.4 ± 0.1 *	0.9 ± 0.1 **	3.3 ± 0.2 *	0.6 ± 0.0

The table shows chlorophyll content (chlorophyll a, b, and total (a+b)) and carotenoids of control (C) and ultrasound-treated (US) seedlings under different osmotic potentials (0 to –0.50 MPa). Data are presented as means ± SE (n=3). Asterisks indicate significant differences between treatments for each osmotic potential: *p ≤ 0.05; **p ≤ 0.01. Statistical significance was assessed using independent Student’s t-tests.

### 3.4 Effects of ultrasound treatment on lipid peroxidation in seeds and seedlings

**Figure 3:**
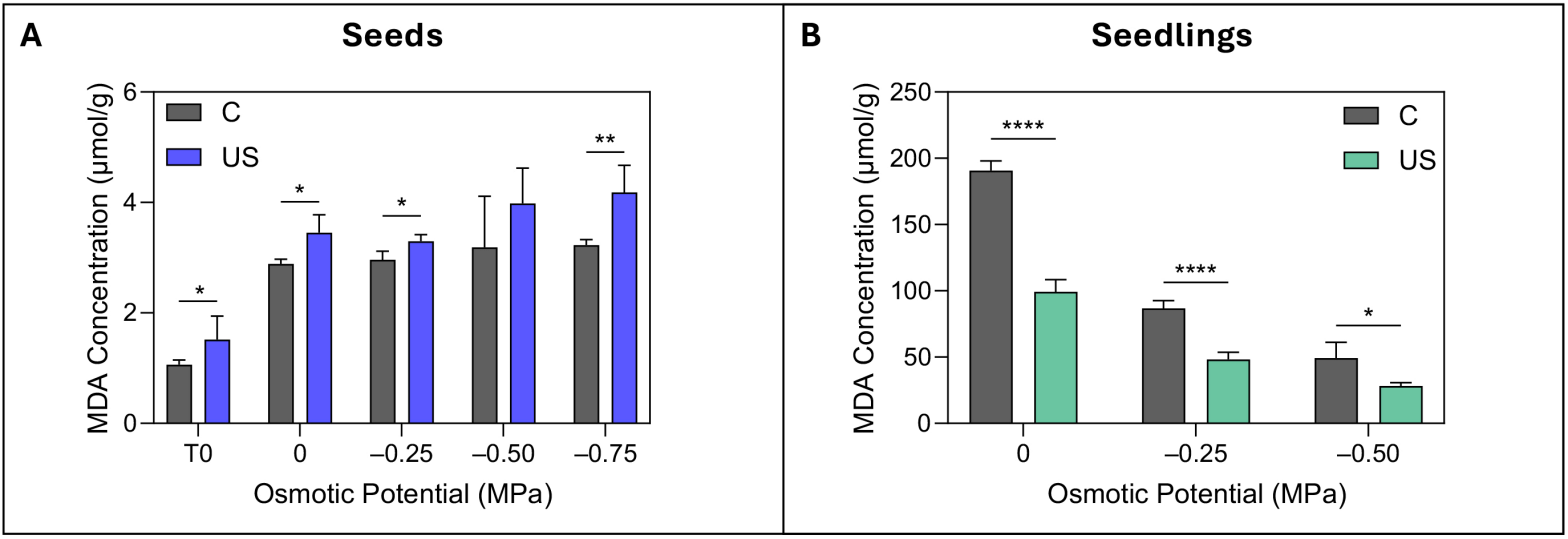
Malondialdehyde content in seeds and seedlings under osmotic stress conditions. MDA concentration (µmol/g) in control (C) and ultrasound-treated (US) seeds across osmotic potentials (0 to –0.75 MPa), with T_0_ indicating seeds prior to germination **(A)**. MDA concentration in seedlings under osmotic stress from 0 to –0.50 MPa **(B)**. Data are presented as means ± SE (n = 3). Asterisks indicate significant differences between treatments for each osmotic potential: *p ≤ 0.05; **p ≤ 0.01; ****p ≤ 0.0001. Statistical significance was assessed using independent Student’s t-tests.

Analysis of lipid peroxidation in seeds indicated that ultrasound-treated seeds consistently exhibited higher MDA concentrations (**Figure 3A**). The increase in MDA content became increasingly pronounced as osmotic stress intensified, reaching a 31% rise at –0.75 MPa. Even under non-stressed conditions (T_0_ and 0 MPa) and mild stress (–0.25 MPa), seeds treated with ultrasound demonstrated significantly elevated MDA levels, while the MDA concentration in control seeds remained stable across the osmotic potentials.

In contrast, seedlings from ultrasound-treated seeds exhibited a markedly different response, with the treatment significantly reducing MDA accumulation. The greatest reduction occurred under non-stress conditions (0 MPa), with MDA levels in seedlings nearly 48% lower than those of the controls. This effect persisted under osmotic stress, with seedlings from treated seeds showing significantly lower MDA levels at both –0.25 and –0.50 MPa, reduced by up to 44% (**Figure 3B**).

### 3.5 Effects of ultrasound treatment on enzymatic activity in seeds and seedlings

**Figure 4:**
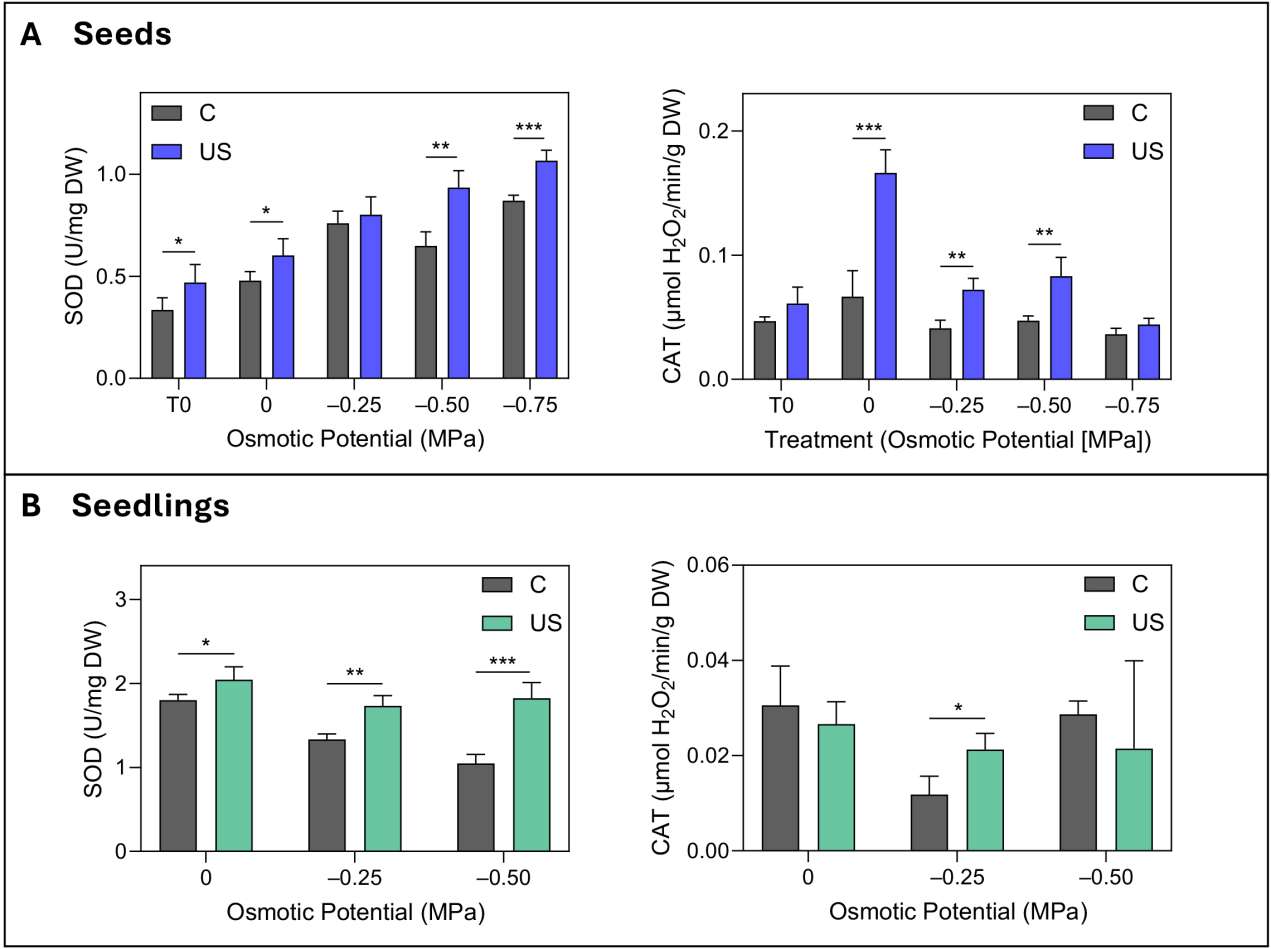
Enzymatic activity in seeds **(A)** and seedlings **(B)** under osmotic stress conditions. SOD (U/mg DW) and CAT (μmol H_2_O_2_/min/g DW) activities in control (C) and ultrasound-treated (US) seeds across osmotic potentials (0 to –0.75 MPa) and in seedlings from 0 to –0.50 MPa. T_0_ represents the seed before the germination process. Data are presented as means ± SE (n=3). Asterisks indicate significant differences between treatments for each osmotic potential: *p ≤ 0.05; **p ≤ 0.01; ***p ≤ 0.001. Statistical significance was assessed using independent Student’s t-tests.

The enzymatic activities of SOD and CAT in seeds demonstrated a coordinated increase in response to ultrasound treatment (**Figure 4A**). Ultrasound-treated seeds exhibited a consistent rise in SOD activity from T_0_ to –0.75 MPa, with the most pronounced difference recorded at –0.50 MPa osmotic potential, showing an increase of 45%. CAT activity remained constant in the control seeds across the osmotic potentials, whereas ultrasound treatment enhanced the activity by 148% in unstressed seeds (0 MPa) and by up to 77% in stressed seeds (–0.25 and –0.50 MPa) (**Figure 4A**).

The enzymatic activity of SOD in seedlings showed a positive response to ultrasound treatment at all osmotic stress levels. The most significant increase was observed at the highest stress condition, with an improvement of 73%, while at 0 MPa the increase was nearly 14%. CAT activity showed a more nuanced response, with a significant 75% increase observed due to ultrasound treatment exclusively at –0.25 MPa stress level (**Figure 4B**).

### 3.6 Effects of ultrasound treatment on antioxidant response

**Figure 5:**
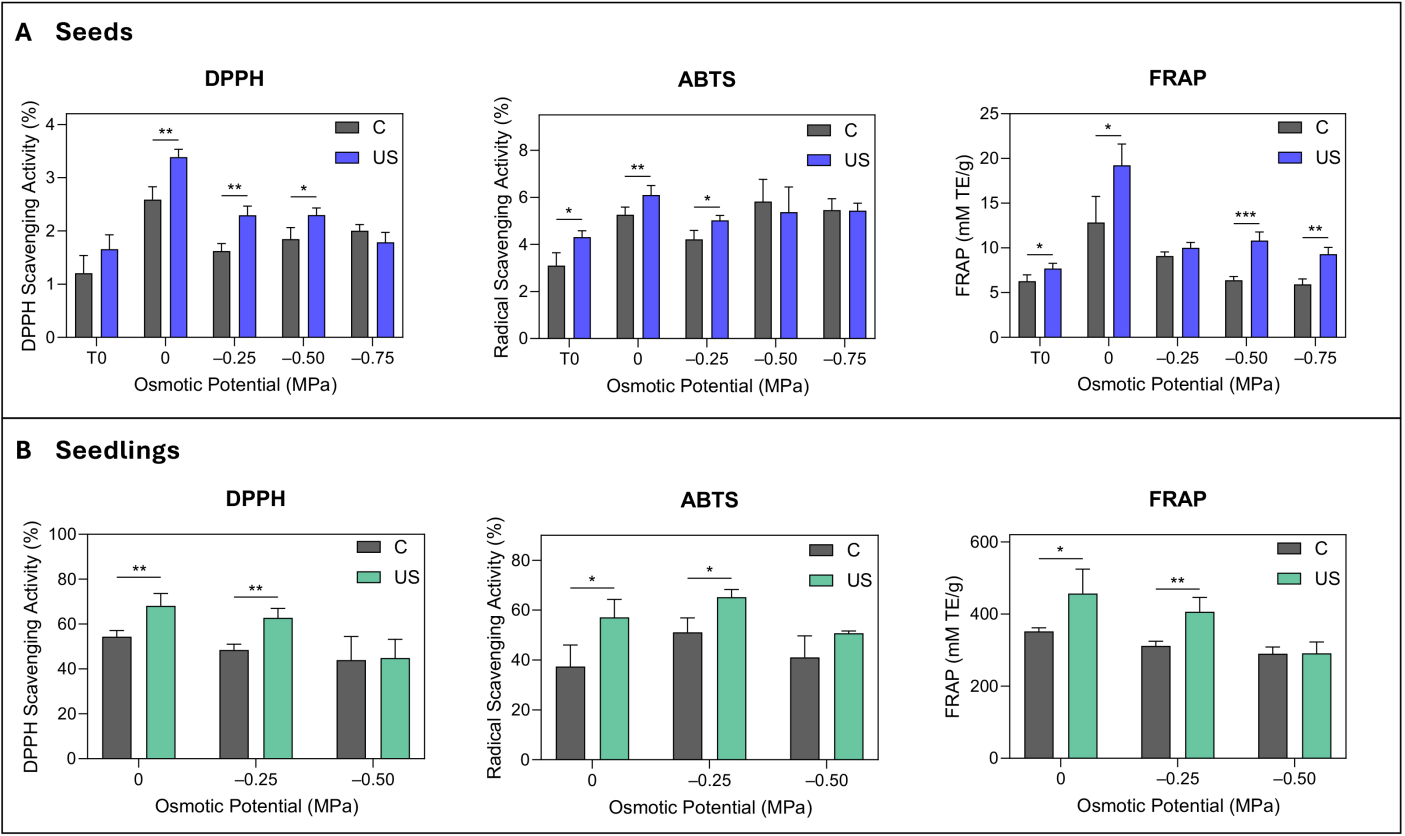
Effect of ultrasound treatment on antioxidant activity in seeds and seedlings under osmotic stress. Antioxidant activity in control (C) and ultrasound-treated (US) seeds measured by DPPH radical scavenging activity (%), ABTS radical scavenging activity (%), and FRAP (mM TE/g) across osmotic potentials (0 to –0.75 MPa), with T_0_ indicating seeds prior to germination **(A)**. Antioxidant activity in seedlings assessed by DPPH, ABTS, and FRAP under osmotic stress from 0 to –0.50 MPa **(B)**. Data are presented as means ± SE (n=3). Asterisks indicate significant differences between treatments for each osmotic potential: *p ≤ 0.05; **p ≤ 0.01; ***p ≤ 0.001. Statistical significance was assessed using independent Student’s t-tests.

Antioxidant activity in control seeds showed variable responses to increasing osmotic stress, however, ultrasound treatment markedly improved it, with the most pronounced effects occurring under moderate osmotic stress conditions (**Figure 5A**). Ultrasound treatment significantly increased the scavenging activity of DPPH (0 to –0.50 MPa) and ABTS (T_0_ to –0.25 MPa) by up to 41% and 39%, respectively. FRAP concentration was greatly enhanced by up to 70% through ultrasound treatment across all conditions (except at –0.50 MPa), spanning from quiescent seeds to those under the highest osmotic stress (–0.75 MPa). The antioxidant activity of control seedlings remained unaffected by the different osmotic pressures, but ultrasound treatment increased the activity in the three assays with a variation of up to 53% and 30% at 0 and –0.25 MPa, respectively (**Figure 5B**).

### 3.7 Correlation analysis of biochemical and physiological traits in seeds and seedlings

**Figure 6:**
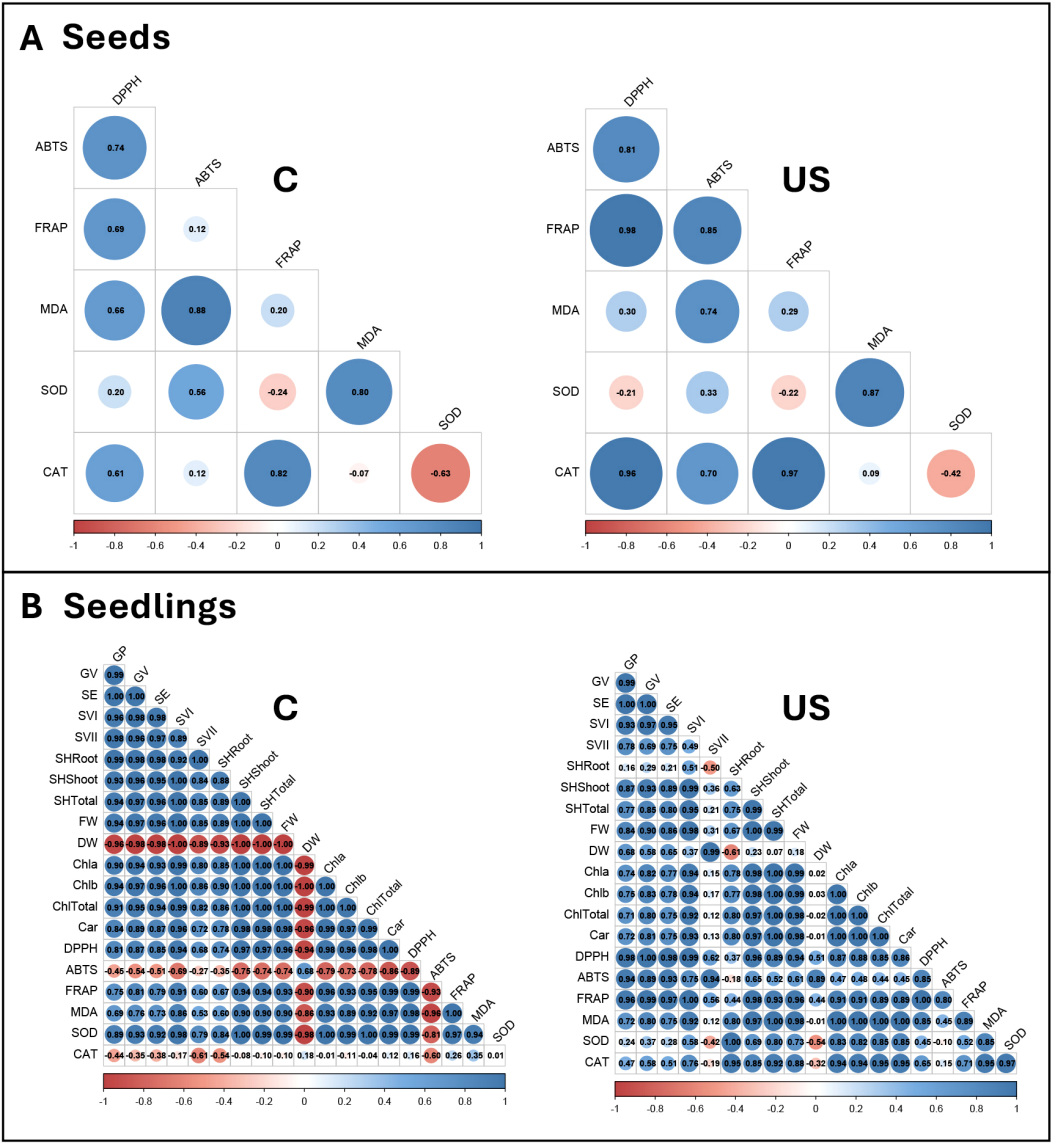
Correlation matrices of biochemical and physiological parameters in tomato seeds **(A)** and seedlings **(B)** under control **(C)** and ultrasound (US) treatments. The size and color intensity of circles represent the strength of correlations, with blue indicating positive correlations and red indicating negative correlations. Values range from -1 (strong negative correlation) to 1 (strong positive correlation). Parameters measured include: antioxidant enzymes (SOD, superoxide dismutase; CAT, catalase), oxidative stress marker (MDA: malondialdehyde), antioxidant capacity (DPPH, ABTS, FRAP), seedling growth parameters (GP, germination percentage; GV, germination value; SE, seedling emergence; SVI-SVII, seedling vigor indices; seedling heights (SHRoot, root; SHShoot, shoot; SHTotal:total (root + shoot)); FW, fresh weight; DW, dry weight), and photosynthetic pigments (Chl _a_, chlorophyll a; Chl _b_, chlorophyll b; Chl _a + b_, total chlorophyll; Car, carotenoids).

The Pearson correlation analysis of seed traits demonstrated a similar pattern in both control and ultrasound samples, with a strong positive correlation observed between antioxidant capacity parameters (DPPH, ABTS, and FRAP) in both treatments (r > 0.81 in ultrasound-treated seeds). SOD exhibited a negative correlation with CAT in both control and ultrasound treatments, with the latter showing lower values (r = –0.63 and r = –0.42, respectively). SOD was found to be negatively correlated with antioxidant capacity, however, in control seeds, this occurred only with FRAP values, while seeds subjected to ultrasound treatment also included the DPPH values (**Figure 6A**). Overall, these relationships indicate a coordinated clustering of antioxidant-related parameters, with ultrasound treatment subtly reshaping the balance between enzymatic and non-enzymatic antioxidant responses in seeds.

The analysis of seedlings indicated that dry weight correlated negatively with physiological, antioxidant (DPPH, FRAP), or enzymatic (SOD, CAT) traits in the control group. Interestingly, ABTS showed a negative correlation with most traits (14), while CAT correlated negatively with indicators of seedling development (**Figure 6B**). In contrast, the seedlings from ultrasound-treated seeds exhibited only occasional negative correlations (e.g., DW with SHshoot, SOD and CAT), highlighting the significant influence of ultrasound seed treatment prior to germination on the physiological and biochemical traits of the seedlings (**Figure 6B**). Collectively, these patterns suggest a clearer separation between growth-related and antioxidant-related trait clusters in ultrasound-derived seedlings, with reduced antagonistic relationships compared with controls.

## 4 Discussion

### 4.1 Ultrasound treatment counteracts the drought effects on seed and seedling traits

Seed germination and early seedling growth are critical stages for plant establishment, during which plants are particularly vulnerable to environmental stresses, especially drought. Drought stress impairs seed germination by restricting water uptake, delaying metabolic processes, and reducing seedling vigor, which affects agricultural productivity worldwide. The severity of these effects depends on the species’ intrinsic tolerance to water deficit (Barnabás et al., 2008; Lobell et al., 2011; Aswathi et al., 2022; Saha et al., 2022). This is particularly relevant for tomato (*Solanum lycopersicum*) cultivation, as drought stress during early developmental stages can significantly impact crop establishment and subsequent yield (Chiwina et al., 2024). Conventional priming methods, including hydropriming, osmopriming, or hormonal priming, have been employed as effective strategies to mitigate drought-induced damage in several species, including soybean (Sintaha et al., 2022), pea (Arafa et al., 2021), cabbage (Yan, 2015), and wheat (Wang et al., 2023), thereby improving their performance. The current study on tomato seeds exposed to drought stress, simulated by decreasing osmotic potentials, demonstrated an enhanced physiological response following ultrasound treatment. The PCA analysis highlighted the positive effects of ultrasound on various physiological traits.

Previous studies have shown that ultrasound treatment can improve seed hydration and germination rates and boost seedling vigor in several crops, such as tomato (Nogueira et al., 2024), soybean (Chen et al., 2023), or barley (Yaldagard et al., 2008). Evidence indicates that the improved water absorption results from mechanical pressure generated by ultrasound-induced acoustic cavitation, which creates micro-fissures in the seed coat, enhancing metabolic activity and germination (Yaldagard et al., 2008; Nogueira et al., 2023; Huang et al., 2024). Studies on castor bean (Babaei et al., 2023) and maize (Gong et al., 2024) seeds have reported beneficial effects on germination under water stress conditions following ultrasound treatment. This may explain the positive effects of ultrasound treatment on tomato seed germination under osmotic stress and how the initial increase in water availability led to a rapid and prolonged impact on seedling development. Similarly, a study on fenugreek seeds exposed to salinity stress, which typically inhibits water uptake and impairs cell growth and division, revealed that ultrasound treatment significantly mitigated these adverse effects. The treatment not only enhanced germination percentage but also extended its benefits to seedling vigor, plumule and root length, as well as fresh and dry weight (El-Sattar and Tawfik, 2023). Even under non-stress conditions, several studies have reported that the ultrasound pre-treatment benefits observed during germination were also carried over to the seedling stage, potentially explaining its advantageous effects under water scarcity conditions (Machikowa et al., 2013; Chiu and Sung, 2014; Alfalahi et al., 2022). It is widely described that water deficit impairs seed germination and seedling growth by: morphologically reducing the seedling length and biomass through inhibition of cell division and elongation; physiologically decreasing water potential and disrupting nutrient transport; biochemically suppressing essential hydrolytic enzymes (e.g., α-amylases, proteases, and lipases) needed for reserve mobilization; and molecularly triggering stress responses through the upregulation of proteins like aquaporins and dehydrins (Yigit et al., 2016; Saha et al., 2022).

The results of the present study, along with other published studies, reveal a positive effect of the ultrasound treatment at these multiple levels. For instance, a significant increase in seedling length and biomass traits was positively correlated with seeds and seedlings performance (Nogueira et al., 2024). A positive effect on seed reserve mobilization was observed, which was correlated with the activity of enzymes involved in starch breakdown (Yaldagard et al., 2008; Miano et al., 2016). Another study revealed that ultrasound modifies seed hormone regulation, particularly affecting key growth regulators such as IAA, GA_3_, and ABA (Huang et al., 2024; Gong et al., 2024). A significant impact of ultrasound treatment on the expression of DNA and protein repair genes in rice seeds was observed (Huang et al., 2024). These findings suggest that ultrasound not only positively influenced the initial stages of plant development but may also activate protective physiological mechanisms responsible for these beneficial effects.

Indeed, the present study showed a significant positive effect of ultrasound on the concentration of chlorophylls under the most severe osmotic stress. It is well established that drought stress reduces photosynthetic activity due to damaged chloroplast structures and impaired chlorophyll biosynthesis, particularly in early enzymatic stages, directly through limiting CO_2_ diffusion and metabolic disruption and indirectly through oxidative stress (Chaves et al., 2009; Pinheiro and Chaves, 2011). Various seed priming methods, including hormonal and chemical treatments, have shown benefits by enhancing stomatal conductance, protecting against pigment degradation, and boosting the activity of carbon-fixing enzymes, ultimately leading to improved photosynthetic efficiency and biomass production (Eisvand et al., 2010; Nazari et al., 2020; Lotfi et al., 2020). While these priming techniques are well documented, ultrasound treatment of seeds has consistently demonstrated comparable, if not superior, benefits, paving the way for a sustainable seed treatment approach that is particularly valuable in drought-prone agricultural regions.

### 4.2 Ultrasound treatment alleviates oxidative damage by improving antioxidant and enzymatic defenses

Drought stress triggers complex cellular disruptions by inducing osmotic stress and generating excessive reactive oxygen species (ROS), leading to oxidative damage in plant cells. The accumulation of ROS, particularly hydrogen peroxide (H_2_O_2_) and superoxide anions (O•_2_^−^), causes severe damage to cellular components by lipid peroxidation, protein degradation, carbohydrate oxidation and DNA fragmentation (Zhang et al., 2015; Chakma et al., 2021; Seleiman et al., 2021; Saha et al., 2022). Additionally, under water deficit conditions, plants experience reduced membrane stability, chlorophyll degradation and increased electrolyte leakage (Wang et al., 2019; Seleiman et al., 2021).

The accumulation of malondialdehyde (MDA), an end-product of membrane lipid degradation, serves as a key oxidative stress-induced cellular damage marker in both seeds and seedlings. In a study of rice seeds subjected to drought stress induced by PEG-6000, an increase in the MDA content was observed, which reduced seed quality and suppressed germination (Liu et al., 2019). Unlike previous studies, our results for tomato seeds showed stable MDA content in the control group despite increasing drought stress, maintaining levels similar to those in non-stressed conditions. However, ultrasound-treated seeds promoted a significant increase in MDA levels at almost all tested osmotic potentials. This may be due to membrane damage caused by ultrasound-induced mechanical stimulation, as reported by Huang et al. (2024) and Alfalahi et al. (2022), which noted disruption in the structure of cellular membranes in seeds, potentially triggering the production of ROS. This effect was previously demonstrated in another study, which revealed a marked increase in MDA content following ultrasound treatment of soybean seeds. The authors argue that ultrasound exposure induces oxidative stress, subsequently triggering the production of isoflavones through the regulation of genes involved in the phenylpropanoid metabolic pathway, thereby improving the antioxidant activities of soybean sprouts (Qiao et al., 2024). This is supported by results observed under T_0_ conditions, where seed samples analyzed immediately after ultrasound treatment (without germination or drought stress) showed higher levels of lipid peroxidation. Studies concerning seedlings of various species, including pea, maize, and rapeseed, have shown that water scarcity increases lipid peroxidation, evidenced by the accumulation of MDA, however, treatments with different priming methods, such as hormonal priming (gibberellic acid and melatonin) and biological priming (*Bacillus thuringiensis*), effectively reduced MDA content in the seedlings (Khan et al., 2020; Arafa et al., 2021; Muhammad et al., 2023). In the present study, MDA content in tomato seedlings decreased with increasing osmotic stress, aligning with findings from previous research. Notably, seedlings grown from ultrasound-treated seeds exhibited a significant reduction in MDA levels, reaching up to 44%. As previously reported, the increased activity and concentration of antioxidant defenses likely enhanced ROS scavenging, promoting membrane stability and preventing excessive MDA accumulation (Qiao et al., 2024). A study on naturally aged seeds of Russian wildrye subjected to ultrasound treatment showed a reduction in MDA levels in the seedlings. The authors highlighted the potential of sonication to restore membrane integrity and inhibit oxidative enzymes in seedlings, including peroxidase (POD) and lipoxygenase (Liu et al., 2016). This effect may contribute to the observed improvement in seedling vigor under osmotic stress conditions in our work.

When exposed to abiotic stresses, plants activate defense mechanisms to scavenge ROS, reducing lipid peroxidation and MDA accumulation, thereby protecting cell membranes. Plants possess other mechanisms to cope with stress tolerance, increasing the production of non-enzymatic antioxidants (phenolics, flavonoids, among others), upregulating antioxidant enzymes, and activating key pathways (e.g., the ascorbate-glutathione cycle), helping maintain osmotic balance and redox homeostasis. However, natural defense mechanisms alone are often insufficient, as the severity of drought impacts varies with crop type, growth stage, and environmental conditions (Huang et al., 2019; Ahmad et al., 2020; Seleiman et al., 2021). SOD and CAT play a fundamental role against oxidative stress. SOD facilitates the transformation of superoxide anions (O_2_•^−^) into hydrogen peroxide (H_2_O_2_) and CAT completes the detoxification process by converting hydrogen peroxide into harmless water and molecular oxygen (Ighodaro and Akinloye, 2018). Previous studies on aged seeds of soybean, tall fescue, and Russian wildrye reported higher enzymatic activity of SOD, CAT, peroxidase (POD) and acetaldehyde dehydrogenase (ALDH) following ultrasound treatment (and the corresponding enzyme-coding genes), collectively contributing to enhanced germination performance of these species (Liu et al., 2016; Huang et al., 2024). The present study demonstrated that ultrasound treatment enhanced SOD activity in both seeds and seedlings under nearly all tested osmotic potentials by up to 45%. While drought stress did not alter CAT activity in control samples, ultrasound treatment significantly increased CAT activity in both seeds and seedlings under osmotic stress conditions.

Additionally, the present study showed a significant increase in antioxidant activity across different osmotic stresses in both seeds and seedlings promoted by ultrasound, which is positively associated with increased seed germination and seedling vigor under water-stressed conditions. To the best of our knowledge, this is the first study to explore the use of ultrasound in modulating the enzymatic and non-enzymatic antioxidant capacity of seeds subjected to osmotic stress. Similarly, other studies have reported identical findings under different experimental conditions. For example, soybean seeds demonstrated significant improvements in germination percentage, sprout length, and antioxidant activity after ultrasound treatment (Qiao et al., 2024). Other studies conducted on oat and black highland barley seeds revealed an increased concentration of total phenolic and flavonoid compounds after ultrasound treatment. Indeed, the literature supports the idea that ultrasound treatment serves as an abiotic stressor, activating plant defense mechanisms that enhance the scavenging activity of reactive oxygen species (ROS) (Ding et al., 2019; Zhang et al., 2023).

## 5 Conclusion

This study indicates the significant potential of ultrasound-assisted seed treatment as an innovative and sustainable strategy to mitigate the adverse effects of drought stress on tomato seed germination and seedling development. The findings reveal that ultrasound treatment noticeably enhances germination rates, seedling vigor, and biomass accumulation under osmotic stress conditions simulated by D-mannitol, with notable improvements in germination percentage (up to 34% at –0.75 MPa) and seedling emergence (up to 36% at –0.50 MPa). Furthermore, ultrasound treatment boosts physiological and biochemical defenses, increasing chlorophyll content (up to 50% at –0.50 MPa) and reducing lipid peroxidation in seedlings, with MDA levels dropping by up to 48% across all conditions. Notably, in both seeds and seedlings, ultrasound enhanced enzymatic antioxidant activities and non-enzymatic antioxidant capacity by up to 77% and 70%, respectively, across various osmotic potentials. These improvements collectively indicate that ultrasound mitigates oxidative damage caused by drought and may offer a scalable, eco-friendly alternative to traditional priming methods.

The differential responses observed between seeds and seedlings underscore the multifaceted impact of ultrasound, with treated seeds exhibiting higher MDA levels, indicative of initial mechanical stress, yet yielding seedlings with superior stress tolerance and growth performance. This suggests that ultrasound induces an adaptive defense mechanism that stabilizes cellular membranes and reduces oxidative stress over time. Further research is needed to fully elucidate the underlying molecular mechanisms, including direct ROS quantification, gene expression analysis and photosynthetic activity. In parallel, optimization of treatment protocols across crops and stress conditions are needed, while also studying the long-term priming dehydration reproducibility. Nevertheless, these results highlight ultrasound technology as a promising tool to enhance crop resilience and support sustainable agricultural productivity in the face of escalating climate challenges.

## Data Availability

All datasets presented in this study are included in the article/**Supplementary Material**.
