# Supplementary material for: Ultrasound treatment enhances tomato drought resistance from germination to seedling stage
Source: Front Plant Sci. 2026 Feb 10;17:1738812. doi: 10.3389/fpls.2026.1738812 (PMC12930468; doi:10.3389/fpls.2026.1738812)
Supplement: Supplementary file 1 [file DataSheet1.docx]

Supplementary Data


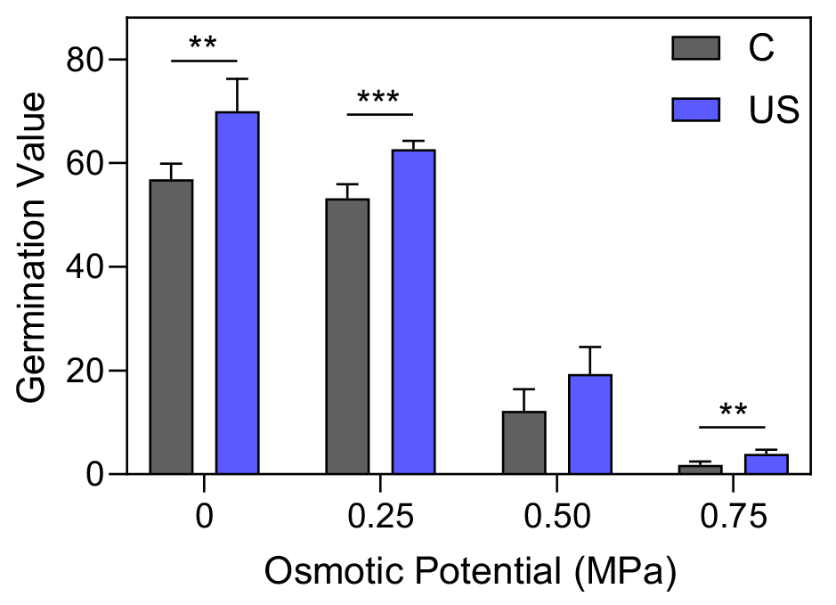


**Fig. S1** Effects of ultrasound treatment on germination value of seeds under different osmotic stress (0 to −0.75 MPa). Data are presented as means ± SE (n=3). Asterisks indicate significant differences between treatments for each osmotic potential: *p < 0.05; **p < 0.01; ***p < 0.001. Statistical significance was assessed using independent Student's t-tests.


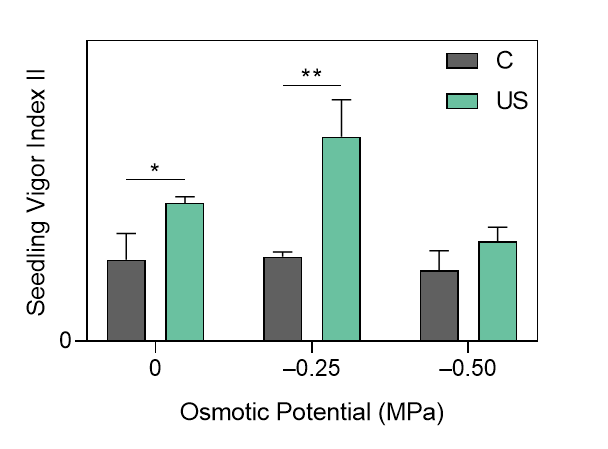


**Fig. S2** Effects of ultrasound treatment on seedling vigor index II on seedlings from ultrasound-treated seeds under different osmotic stress (0 to −0.50 MPa). Data are presented as means ± SE (n=3). Asterisks indicate significant differences between treatments for each osmotic potential: *p < 0.05; **p < 0.01; ***p < 0.001. Statistical significance was assessed using independent Student's t-tests.
